# M2 Macrophages Promote PDGFRβ^+^ Pericytes Migration After Spinal Cord Injury in Mice *via* PDGFB/PDGFRβ Pathway

**DOI:** 10.3389/fphar.2021.670813

**Published:** 2021-04-15

**Authors:** Ziyu Li, Meige Zheng, Shuisheng Yu, Fei Yao, Yang Luo, Yanchang Liu, Dasheng Tian, Li Cheng, Juehua Jing

**Affiliations:** Department of Orthopaedics, The Second Hospital of Anhui Medical University, Hefei, China

**Keywords:** spinal cord injury, M2 macrophages, PDGFRβ^+^ pericytes, PDGFRβ, fibrotic scar

## Abstract

Platelet derived growth factor receptor β positive (PDGFRβ^+^) pericytes form fibrotic scar, which prevents axonal regeneration after spinal cord injury (SCI). However, the mechanism by which PDGFRβ^+^ pericytes migrate to the injury core is unclear. Here, we investigated the effect and mechanism of macrophages polarization on PDGFRβ^+^ pericytes migration after SCI. Macrophages were closely related to the spatiotemporal distribution of PDGFRβ^+^ pericytes in the injury core at 3, 7, and 14 days postinjury (dpi). Macrophages appeared M2 polarization at 3 and 7 dpi while M1 polarization at 14 dpi. The expression of platelet derived growth factor B (PDGFB) was significantly increased after SCI and after macrophages M2 polarization. The promoting effect of exogenous PDGFB and M2 macrophages conditioned medium on PDGFRβ^+^ pericytes migration could be blocked by SU16f, a PDGFRβ specific inhibitor. These findings indicate that M2 macrophages can secrete PDGFB acting on PDGFRβ to promote PDGFRβ^+^ pericytes migration, which can be blocked by a PDGFRβ specific inhibitor SU16f. The PDGFB/PDGFRβ pathway is a promising new target for the treatment of SCI.

## Introduction

Spinal cord injury (SCI) is a devastating injury resulting in severe and sustained sensory and motor dysfunction in patients, and there is no effective clinical treatment (Selvarajah et al., 2015; Bellver-Landete et al., 2019; Kobayakawa et al., 2019). In the acute phase of SCI, physical trauma leads directly to the disruption of the blood–spinal cord barrier and bleeding, followed by the infiltration of blood-derived macrophages (David and Kroner, 2011). Oxidative stress caused by ischemia-reperfusion injury and a large number of inflammatory factors released by macrophages further aggravate inflammation, which contributes to neuronal and glial apoptosis (Hausmann, 2003; Muradov et al., 2013; Tran et al., 2018). In the subacute phase of SCI, platelet derived growth factor receptor β positive (PDGFRβ^+^) pericytes gradually migrate and aggregate to the injury core and deposit extracellular matrix, which surround macrophages and form fibrotic scar (Göritz et al., 2011; Soderblom et al., 2013). Although fibrotic scar prevents the spread of inflammation, it inhibits axonal regeneration in the chronic phase of SCI (Klapka et al., 2005; Göritz et al., 2011; Dias et al., 2018). The removal of fibrotic scar contributes to axonal regeneration suggesting that fibrotic scar may be a target for clinical treatment (Klapka et al., 2005; Dias et al., 2018). However, the mechanisms of PDGFRβ^+^ pericytes migration and extracellular matrix deposition are still unclear.

The clearance of blood-derived macrophages resulted in the reduction of fibrotic scar in the injury core after SCI in mice, suggesting that macrophages may be involved in PDGFRβ^+^ pericytes migration (Zhu et al., 2015a). Moreover, macrophages have different functions due to M1 or M2 polarization in many diseases, which is important for oxidative stress, immune response and homeostasis repair (Kigerl et al., 2009; David and Kroner, 2011; Wang et al., 2015; Wang et al., 2019). It has been shown that platelet derived growth factor (PDGFB) can activate PDGFRβ to regulate pericyte migration in angiopoiesis, fibrosarcoma and retinal tissue (Abramsson et al., 2003; Abramsson et al., 2007; Stratman et al., 2010; Dubrac et al., 2018). However, the effects of macrophages polarization and PDGFB/PDGFRβ pathway on the migration of PDGFRβ^+^ pericytes to the injury core after SCI have not been reported.

In this study, we found that macrophages are closely related to the spatiotemporal distribution of PDGFRβ^+^ pericytes in the injury core at 3, 7, and 14days postinjury (dpi). Macrophages appear M2 polarization at 3 and 7dpi while M1 polarization at 14 dpi. M2 macrophages can secrete PDGFB acting on PDGFRβ to promote PDGFRβ^+^ pericytes migration, which can be blocked by SU16f, a PDGFRβ specific inhibitor. Besides, M2 macrophages can promote PDGFRβ^+^ pericytes to secrete extracellular matrix. These suggested that PDGFB/PDGFRβ pathway is a promising new target for the treatment of SCI.

## Materials and Methods

### Animals and Cells

All animal experiments were approved by the Animal Ethics Committee of Anhui Medical University (Approval No. LLSC20160052). 20–25 g mice were purchased from the Animal Experiment Center of Anhui Medical University. All mice were bred in the Research and Experimental Center of the Second Hospital of Anhui Medical University. The temperature and humidity were controlled in the feeding environment, with a 12 h cycle of light and darkness, and food and water were readily available. Mouse mononuclear macrophage leukemia cells RAW 264.7 were cultured in DMEM (Hyclone, Invitrogen, United States) containing 10% FBS (Gibco, United States), 1% Glutamax and 1% Sodium Pyruvate (Invitrogen, United States), and were kindly provided by Stem Cell Bank, Chinese Academy of Sciences (Shanghai, China). Mouse brain vascular pericytes MBVP were kindly provided by Mingzhou Biological Technology (Ningbo, China), and were cultured in DMEM containing 10% FBS. The cells were cultured at 5% CO_2_ and 37°C. MBVP cells were identified as PDGFRβ^+^ cells *via* immunocytochemistry and could be used as a model of PDGFRβ^+^ pericytes *in vitro*.

### Establishment of Mouse Spinal Cord Compression Injury Model

Each mouse was given water fasting before surgery. The mice were put in prone position after satisfactory anesthesia with isoflurane (induction 4%, maintenance 2%). After shaving the dorsal surface of the mouse and disinfecting the skin, T10 segment of spinal cord was exposed. The T10 spinal cord was clamped with the calibrated Dumont #5 forceps (11252-20, Fine Science Tools, Germany) for 5 s (McDonough et al., 2015). The incisions were sterilized and stitched layer by layer. The mice were routinely treated with anti-infection and auxiliary urination nursing after SCI.

### Establishment of Macrophages Polarization Model and Extraction of Conditioned Medium

RAW 264.7 cells were plated in 6-well plates at a density of 2 × 10^5^/ml and cultured overnight. After serum deprivation for 24 h, RAW 264.7 cells were treated with LPS (100 ng/ml, Beyotime Biotechnology, China) plus IFNγ (20 ng/ml, Beyotime Biotechnology, China) for M1 polarization or IL-4 (20 ng/ml, Beyotime Biotechnology, China) for M2 polarization, and the cells were cultured for 24 h. Then, the cell culture medium was replaced with serum-free DMEM for another 24 h. Supernatant was collected and centrifuged (1200 rpm, 5 min) as conditioned medium including conditioned medium of M0 (CM0), CM1, and CM2. WB and immunocytochemistry were used to identify the macrophage polarization model, and ELISA was used to identify the conditioned medium.

### Immunofluorescence Staining

The mice underwent thoracotomy after satisfactory anesthesia. After cardiac perfusion with PBS and 4% paraformaldehyde (4% PFA, Servicebio, China), spinal cord containing the injured core (5 mm) was removed and fixed in 4% PFA overnight. Then, the tissue blocks were dehydrated, made transparent, dipped in wax, embedded, and sliced to 6 µm thickness serial sections using a microtome (RM2235, Leica, Germany). The sections were deparaffinized and rehydrated in turn. Antigen retrieval was performed using the microwave method, followed by washing with PBS and blocking in 5% donkey serum (SL050, Solarbio, China) in PBS with 0.3% Triton-X100 for 1 h at room temperature. The primary antibodies were incubated at 4°C overnight: Goat anti-PDGFRβ (5 µg/ml, AF1042-SP, R&D Systems, United States), Mouse anti-Mac2 (1:100, GB12246, Servicebio, China), Rabbit anti-iNOS (1:100, AF0199, Affinity, United States) and Rabbit anti-CD206 (1:100, ab64693, Abcam, China). The secondary antibodies were incubated at 37°C in dark for 1 h: Donkey anti-Rabbit Alexa Fluor 488, Donkey anti-Mouse Alexa Fluor 594, Donkey anti-Goat Alexa Fluor 488 and Donkey anti-Goat Alexa Fluor 594 (1:500, A-21206, A-21203, A-11055, A-11058, Invitrogen, United States). Finally, the anti-fluorescence quencher (P0126, Beyotime Biotechnology, China) was used to seal the sections. The representative images were obtained using fluorescence microscope (Axio Scope A1, Zeiss, Germany) by comparing and processing all images of the same light intensity and filtering.

### Immunocytochemistry Staining

Cells were fixed with 4% PFA for 10–15 min and blocked for 30 min at room temperature. Primary antibodies were diluted in blocking solution and applied for overnight at 4°C: Goat anti-PDGFRβ (5 µg/ml, AF1042-SP, R&D Systems, United States), Rabbit anti-iNOS (1:100, AF0199, Affinity, United States), Rabbit anti-CD206 (1:100, ab64693, Abcam, China) and Rabbit anti-Laminin (1:100, 23498-1-AP, Proteintech, China). Secondary antibody incubation as shown above, and blue counterstaining (DAPI, C0065, Solarbio, China) showed nuclei. Finally, the anti-fluorescence quencher was used to seal the sections. The representative images were obtained as above. Image J (NIH, United States) was used for quantitative analysis.

### Western Blot Assay

For cell proteins, cells were washed with PBS and lysed using RIPA buffer (P0013B, Beyotime Biotechnology, China) supplemented with Protease Inhibitor Cocktail and Phosphatase Inhibitor (04693124001, 04906845001, Roche, Switzerland). For spinal cord tissue proteins, after cardiac perfusion with PBS, the spinal cord (3 mm) containing the injury core was removed and lysed with lysis buffer mentioned above. The supernatant was collected as total protein. Protein concentrations were determined using bicinchoninic acid (BCA) kit (P0012S, Beyotime Biotechnology, China) and diluted to 1 mg/ml for each sample. 20 μg of protein was denatured at 100°C for 10 min in LDS Sample Buffer (NP0007, Invitrogen, Unites States) before being resolved on SDS-PAGE electrophoresis and transferred onto a Polyvinylidene difluoride (PVDF) membranes. Blots were blocked in 5% nonfat milk and probed with primary antibodies against at 4°C overnight: Rabbit anti-iNOS (1:2000, AF0199, Affinity, United States), Rabbit anti-CD206 (1:1,000, ab64693, Abcam, China), Goat anti-PDGFRβ (0.25 µg/ml, AF1042-SP, R&D Systems, United States), Mouse anti-PDGFB (1:500, SC-365805, Santa Cruz, United States), Mouse anti-PDGFD (1:500, 14075-1-AP, Proteintech, China), Rabbit anti-Laminin (1:1000, 23498-1-AP, Proteintech, China), Mouse anti-β-Tubulin (1:10000, T0023, Affinity, Unites States) and Mouse anti-GAPDH (1:1000, ab8245, Abcam, China). The membranes were incubated with secondary antibodies for 1 h at room temperature: Goat anti-Mouse (1:10000, A4416, Sigma, United States), Goat anti-Rabbit (1:10000, A0545, Sigma, United States) or Rabbit anti-Goat (1:10000, A8919, Sigma, United States). The protein band signals were obtained using Tanon 5200 system (Tanon, China). Image J (NIH, United States) was used for quantitative analysis.

### Enzyme-Linked Immunosorbent Assay (ELISA)

The PDGFB ELISA kit (KE10034, Proteintech, China) was used to detect the concentration of PDGFB in the conditioned medium of different polarized macrophages. The supernatant was collected and centrifuged (1200 rpm, 5 min) for detection immediately, according to the kit’s instruction.

### Scratch Test

The marker pen was used to draw 3 horizontal lines evenly on the back of the 6-well plate at intervals of 1 cm. MBVP cells were plated in 6-well plates at a density of 2 × 10^5^/ml and cultured until the cells covered the entire plate bottom. The wound was created using a sterile 200 μl pipette tip. After washing off the exfoliated cells, macrophages conditioned medium, serum-free DMEM containing recombinant mouse PDGFB protein (10 ng/ml, NBP2-53416, Novus, United States) or serum-free DMEM containing SU16f (10 μm, 3304, R&D Systems, United States) were added to the 6-well plate to regulate MBVP cells migration for 72 h. The pictures of scratch wounds were taken at 0 and 72 h and assessed using the Image J software.

### Transwell Test

The 24-well plate containing 8 μm chamber (Corning, United States was used to detect the migration ability of cells. MBVP cells were inoculated to the upper chamber at a density of 1 × 10^6^/100 μl. According to the experimental requirements, macrophages conditioned medium or PDGFB (10 ng/ml, NBP2-53416, Novus, United States) were added to the lower chamber to regulate MBVP cells migration, or SU16f (10 μm, 3304, R&D Systems, United States) was added to the upper chamber to block PDGFRβ. After 24 h in an incubator at 37°C and 5% CO_2_, a cotton swab was used to wipe off the cells in the inner layer of chamber. After washing with ddH_2_O water twice, the chambers were fixed by 4% PFA at room temperature for 20 min. After washing with ddH_2_O water twice, crystal violet (C0121, Beyotime Biotechnology, China) staining was performed for 15 min. The cells were observed under the microscope and counted.

### Statistical Analysis

The data were presented as means ± standard deviation. Multiple-group comparisons were statistically analyzed with one-way analysis of variance (ANOVA) followed by the Tukey method or nonparametric test using the Prism 8.3.0 software (GraphPad, United States). *p <* 0.05 was considered significant.

## Results

### The Spatiotemporal Distribution of Macrophages and PDGFRβ^+^ Pericytes is Closely Related After Spinal Cord Injury

**FIGURE 1:**
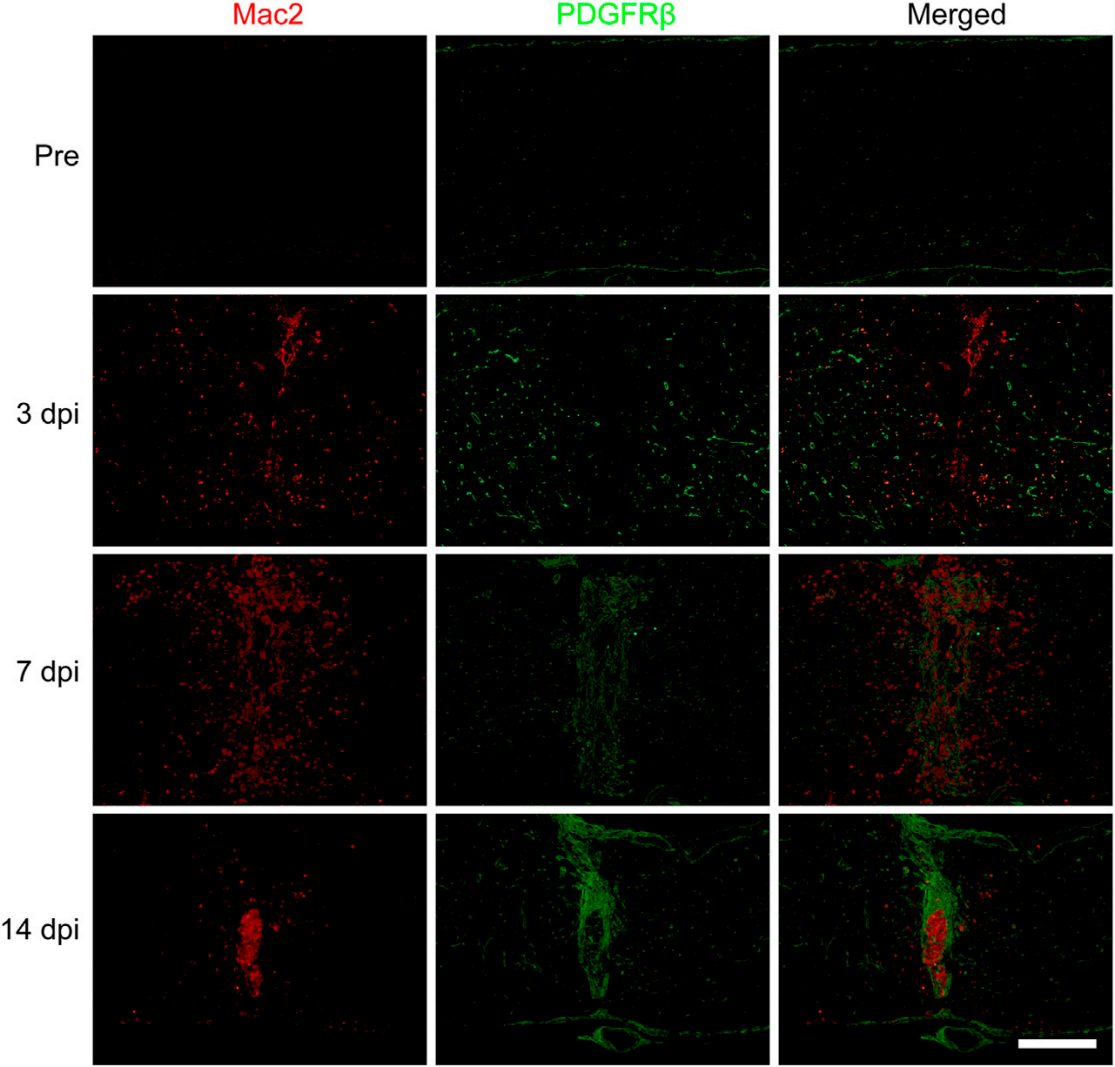
Spatiotemporal distribution of macrophages and PDGFRβ^+^ pericytes after SCI in mice. Immunofluorescence staining was used to detect the distribution of macrophages (Mac2^+^, red) and PDGFRβ^+^ pericytes (PDGFRβ^+^, green) before (Pre) and at 3, 7, and 14 dpi in the injury core (*n* = 3 per group). Representative fluorescence images were presented. Scale bars, 500 μm.

To further confirm the spatiotemporal distribution of macrophages and PDGFRβ^+^ pericytes after SCI, the goat anti-PDGFRβ antibody was used to specifically label PDGFRβ^+^ pericytes while the mouse anti-Mac2 antibody was used to specifically label blood-derived macrophages rather than microglia (Wang et al., 2015). The immunofluorescence staining showed that there was no obvious aggregation of macrophages and PDGFRβ^+^ pericytes in the normal spinal cord. Macrophages and PDGFRβ^+^ pericytes were scattered at the injury core at 3 dpi while significantly aggregated at the injury core at 7 dpi. Finally, PDGFRβ^+^ pericytes surrounded macrophages to form fibrotic scar at 14 dpi (Figure 1). During 3–14 dpi, PDGFRβ^+^ pericytes gradually migrated and aggregated to the injury core, and the spatiotemporal distribution between macrophages and PDGFRβ^+^ pericytes was closely related (Figure 1). These suggested that 3–7 dpi is a critical period for PDGFRβ^+^ pericytes to migrate and aggregate to the injury core, and macrophages may be involved in the migration of PDGFRβ^+^ pericytes after SCI.

### Macrophages Appear M1 or M2 Polarization After Spinal Cord Injury

**FIGURE 2:**
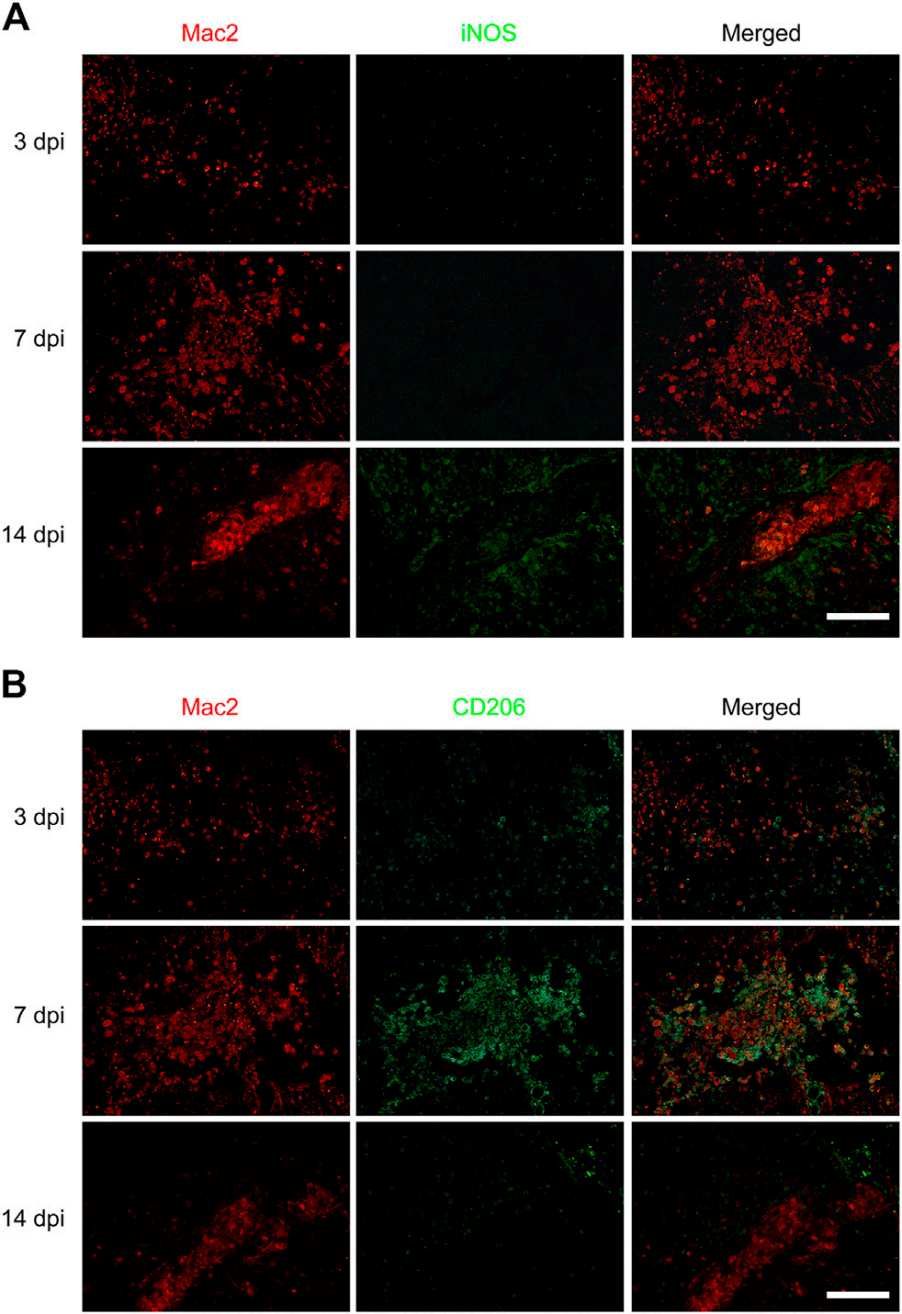
Macrophages polarization after SCI in mice. Immunofluorescence staining was used to detect Mac2^+^ (red) and iNOS^+^ (green) M1 macrophages in **(A)** or Mac2^+^ (red) and CD206^+^ (green) M2 macrophages in **(B)** at 3, 7, and 14 dpi in the injury core (*n* = 3 per group). Representative fluorescence images were presented. Scale bars, 100 μm.

To investigate the effect of macrophages polarization on PDGFRβ^+^ pericytes migration, anti-iNOS antibody and anti-CD206 antibody were used to specifically label M1 and M2 macrophages respectively to observe the macrophages polarization after SCI. The immunofluorescence staining showed that the macrophages (Mac2^+^) were mainly of type M2 with CD206^+^ and iNOS^−^ in injury core at 3 and 7 dpi (Figure 2B) while the macrophages were mainly of type M1 with iNOS^+^ and CD206^−^ in injury core at 14 dpi (Figure 2A). It was interesting to note that macrophages were mainly type M2 in the critical period of 3–7 dpi during when PDGFRβ^+^ pericytes migrated and aggregated to the injury core (Figures 1, 2B). These suggested that M2 macrophages may be involved in the migration of PDGFRβ^+^ pericytes after SCI.

### Identification of PDGFRβ^+^ Pericytes and Macrophages Polarization *in Vitro*

**FIGURE 3:**
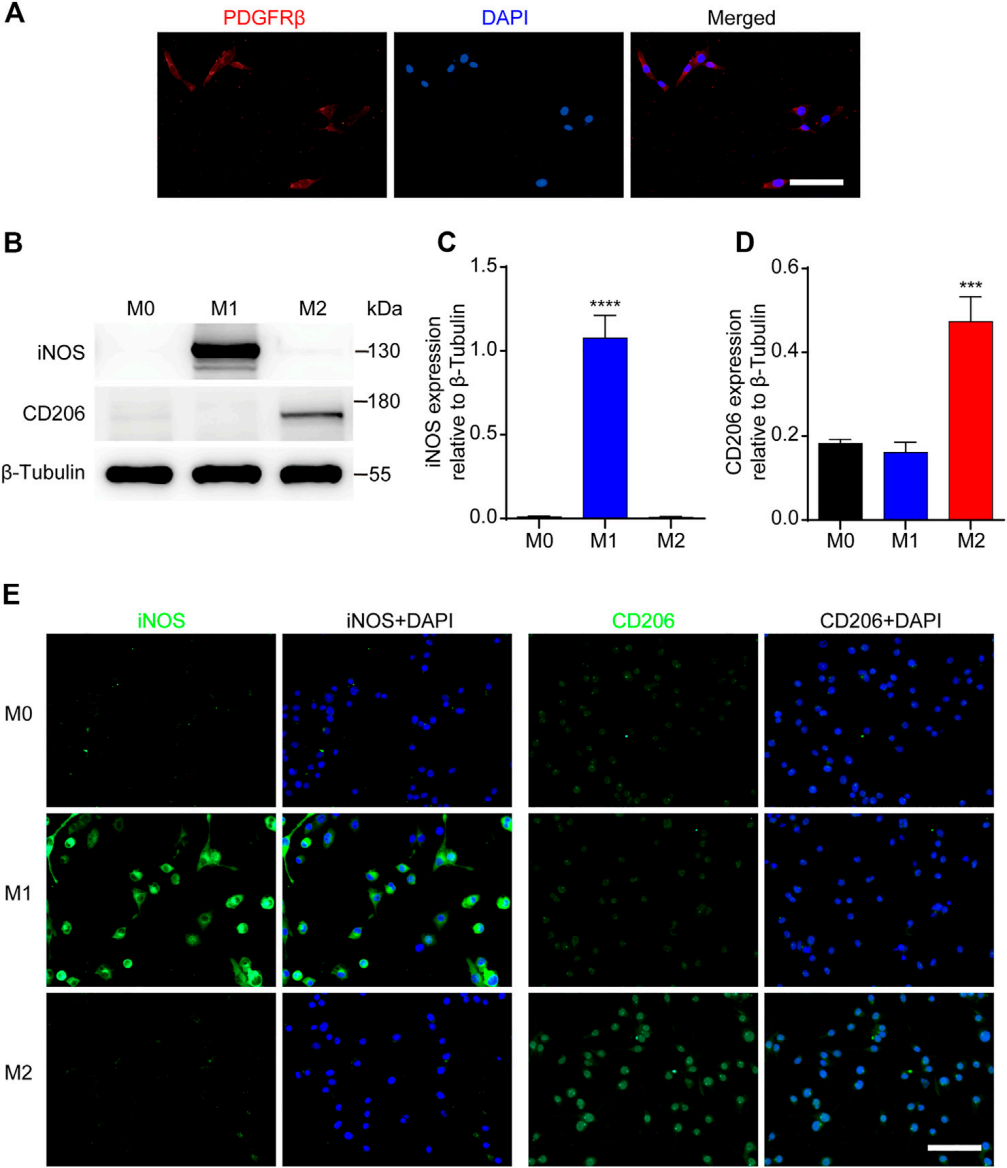
Identification of PDGFRβ^+^ pericytes and polarized macrophages *in vitro*. **(A)** Immunocytochemistry staining was used to detect PDGFRβ^+^ (green) and DAPI-stained nuclei (blue) in MBVP cells. **(B)** Western blot was used to detect the expression levels of M1 (iNOS) or M2 (CD206) polarization markers in RAW 264.7 cells after polarization treatment. **(C, D)** Quantitative analysis of iNOS and CD206 expression. The blots (*n* = 5 per group) were quantified by a densitometric method using the ImageJ software. The results were expressed as mean ± SD. ****p < 0.0001 (CM1 vs. other groups) in **(C)**; ***p < 0.001 (CM2 vs. other groups) in **(D)**. **(E)** Representative fluorescence images of iNOS and CD206 in RAW 264.7 cells after polarization treatment, DAPI stained the nuclei. Scale bars, 50 μm.

To further investigate the effect of M2 macrophages on PDGFRβ^+^ pericytes migration, RAW 264.7 cells were used as the blood-derived macrophages model and MBVP cells as the PDGFRβ^+^ pericytes model *in vitro*. The results of immunocytochemistry suggested that MBVP cells were PDGFRβ^+^ cells and could be used as the PDGFRβ^+^ pericytes model *in vitro* (Figure 3A)*.* In addition, we induced and identified M1 or M2 polarization of macrophages according to previous reports (Kigerl et al., 2009; Hiroi et al., 2013; Fan et al., 2020). The results of Western blot showed that compared with other groups, M1 macrophages significantly expressed iNOS while M2 macrophages significantly expressed CD206 (*p* < 0.0001 for iNOS, *p* < 0.001 for CD206, Figures 3B–D). Besides, immunocytochemistry was used to further confirm the reliability of macrophages polarization model. The results showed that compared with the other two groups, the fluorescence intensity of iNOS of M1 macrophages was significantly increased while that of CD206 of M2 macrophages was significantly increased (Figure 3E). These suggested that the PDGFRβ^+^ pericytes model and macrophages polarization model could be used for the follow-up study.

### M2 Macrophages Promote the Migration of PDGFRβ^+^ Pericytes *in Vitro*

**FIGURE 4:**
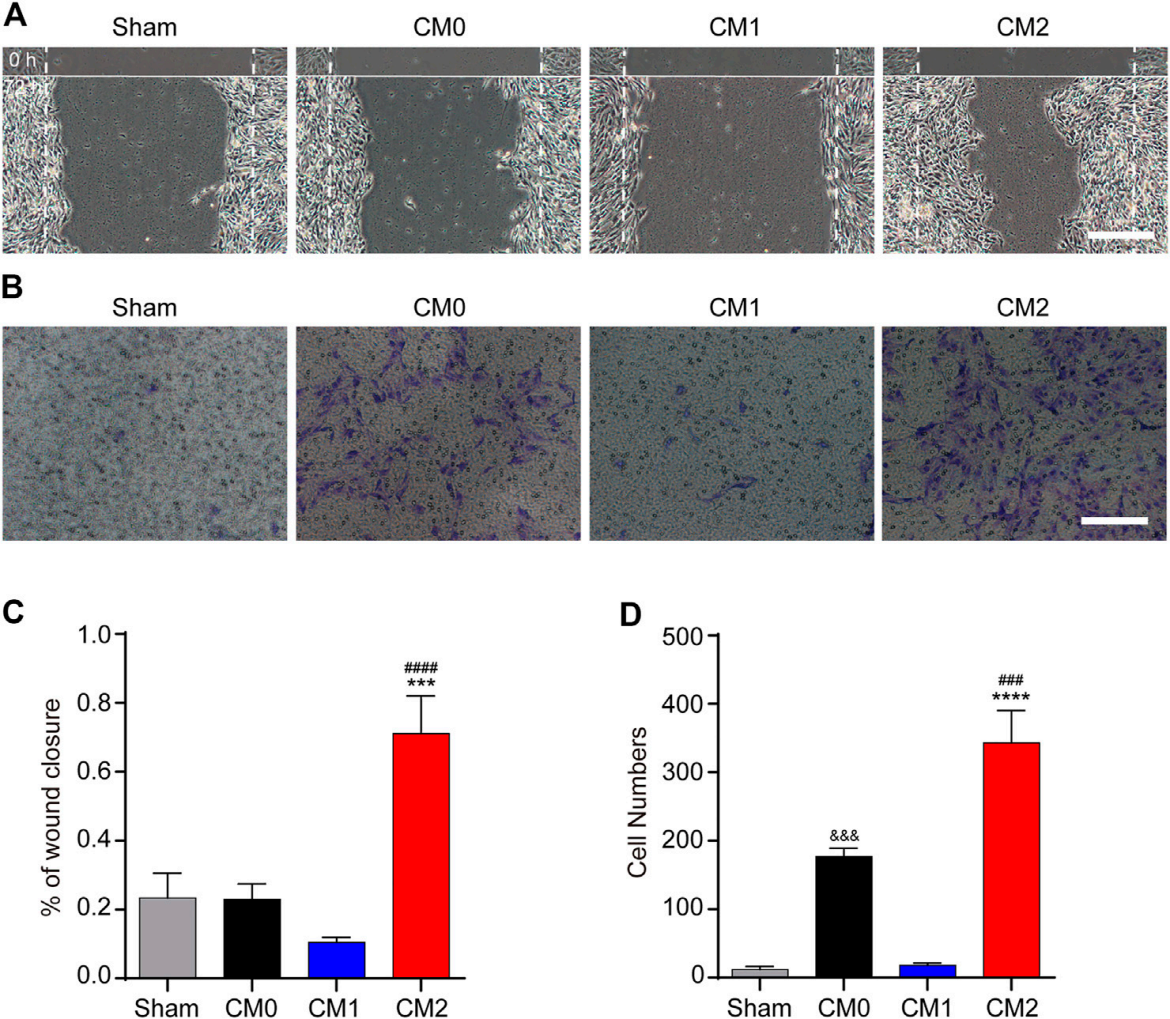
M2 macrophages promote the migration of PDGFRβ^+^ pericytes *in vitro*. **(A)** Scratch test was used to detect the migration of PDGFRβ^+^ pericytes after being treated with DMEM (Sham), conditioned medium of M0 macrophages (CM0), conditioned medium of M1 macrophages (CM1), or conditioned medium of M2 macrophages (CM2) for 72 h (*n* = 3 per group). **(B)** Transwell test was used to further detect the migration of PDGFRβ^+^ pericytes after being treated in Sham, CM0, CM1, or CM2 groups for 20 h (*n* = 3 per group) **(C)** Quantitative analysis of the wound closure rate in **(A)**. The results were expressed as mean ± SD. ***p < 0.001 (CM2 vs. Sham and CM0); ^####^ *p* < 0.0001 (CM2 vs. CM1) **(D)** Quantitative analysis of the number of transmembrane cells in **(B)**. The results were expressed as mean ± SD. ****p < 0.0001 (CM2 vs. Sham and CM1); ^###^ *p* < 0.001 (CM2 vs. CM0). ^&&&^ *p* < 0.001 (CM0 vs. Sham and CM1). Scale bars, 100 μm.

To further investigate the effect of macrophages polarization on PDGFRβ^+^ pericytes migration, the conditioned medium of different polarized macrophages (CM0, CM1, and CM2) were extracted for culture of pericytes. The scratch test showed that after adding M2 conditioned medium, the wound closure rate of PDGFRβ^+^ pericytes was significantly higher than that of other groups (*p* < 0.001 or *p* < 0.0001, Figures 4A,C). Besides, transwell test was used to further show that the number of pericytes passing through membrane increased significantly after adding M2 conditioned medium compared with other groups (*p* < 0.001 or *p* < 0.0001, Figures 4B,D). Compared with Sham group and CM1 group, the number of pericytes passing through membrane increased significantly after adding M0 conditioned medium (*p* < 0.001), but this effect was much weaker than M2 conditioned medium. These suggested that M2 macrophages could promote PDGFRβ^+^ pericytes migration *in vitro*, possibly due to the secretion of certain cytokines.

### M2 Macrophages Promote PDGFRβ^+^ Pericytes to Secrete Extracellular Matrix *in Vitro*

**FIGURE 5:**
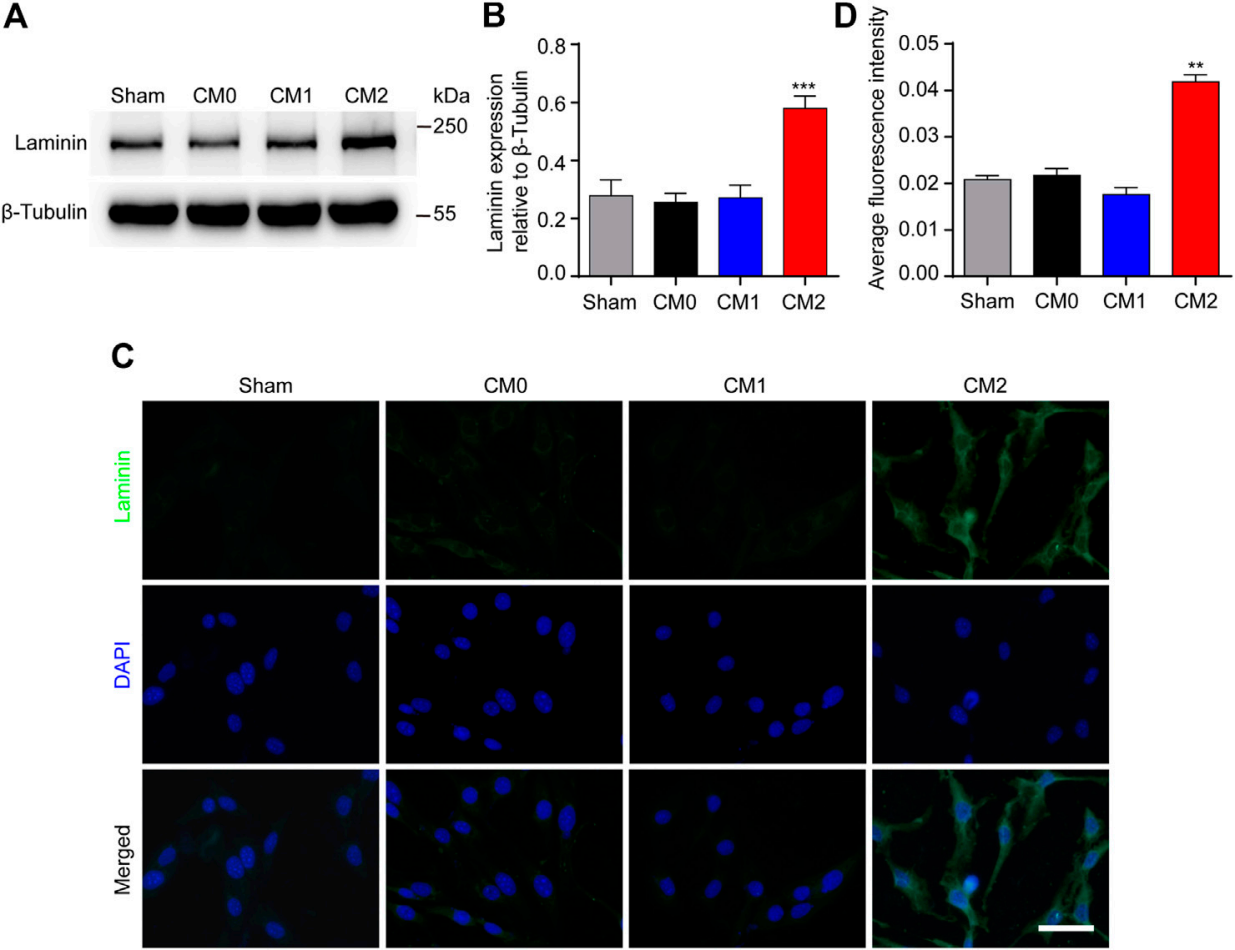
M2 macrophages promote PDGFRβ^+^ pericytes to secrete extracellular matrix Laminin *in vitro*. **(A)** Western blot was used to detect the expression levels of Laminin in PDGFRβ^+^ pericytes after being treated in Sham, CM0, CM1 or CM2 groups for 24 h (*n* = 5 per group). **(B)** Quantitative analysis of Laminin expression. The blots were quantified as previously described. ***p < 0.001 (CM2 vs. other groups). **(C)** Immunocytochemistry staining was used to further confirm the expression levels of Laminin of PDGFRβ^+^ pericytes after being treated in Sham, CM0, CM1 or CM2 groups for 24 h (*n* = 3 per group) **(D)** Quantitative analysis of fluorescence intensity in **(C)**. The results were expressed as mean ± SD. **p < 0.01 (CM2 vs. other groups). Scale bar, 25 μm.

PDGFRβ^+^ pericytes and extracellular matrix including Collagen, Fibronectin and Laminin together surround macrophages to form fibrotic scar after SCI (Soderblom et al., 2013). It has been shown that Collagen and Fibronectin are mainly secreted by PDGFRβ^+^ pericytes after SCI while the main cellular origin of Laminin is unclear (Soderblom et al., 2013; Zhu et al., 2015b). The results of Western blot showed that after adding M2 conditioned medium, the expression level of Laminin in pericytes was significantly higher than that in other groups (*p* < 0.001, Figures 5A,B). Besides, immunocytochemistry was used to further show that the fluorescence intensity of Laminin in pericytes cultured in M2 conditioned medium was significantly higher than that of other groups (*p* < 0.01, Figures 5C,D). These suggested that Laminin may also be mainly secreted by PDGFRβ^+^ pericytes, and M2 macrophages can promote PDGFRβ^+^ pericytes to secrete extracellular matrix *in vitro*.

### The Expression of PDGFB is Significantly Increased After Spinal Cord Injury *in Vivo* and Macrophages M2 Polarization *in Vitro*

**FIGURE 6:**
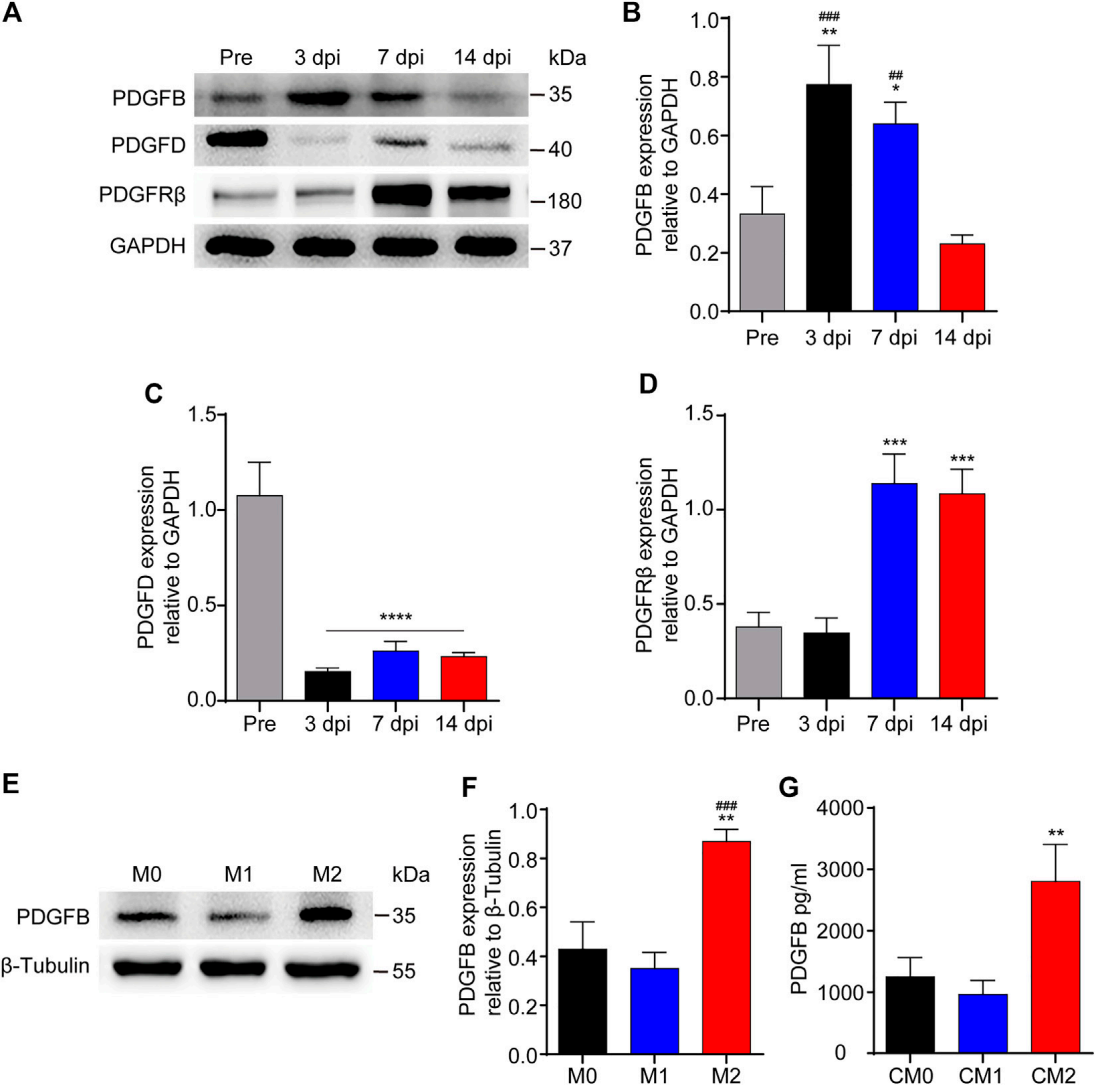
The expression of PDGFB is significantly increased after SCI or macrophages M2 polarization. **(A)** Western blot was used to detect the expression levels of PDGFB, PDGFD and PDGFRβ before (Pre) and at 3, 7, and 14 dpi in the injury core of mice (*n* = 5 per group). **(B)** Quantitative analysis of PDGFB in **(A)**. The blots were quantified as previously described. **p < 0.01, *p < 0.05 (3 and 7 dpi vs. pre); ^###^ *p* < 0.001, ^##^ *p* < 0.01 (3 and 7 dpi vs. 14 dpi). **(C)** Quantitative analysis of PDGFD in **(A)**. ****p < 0.0001 (3, 7, and 14 dpi vs. pre). **(D)** Quantitative analysis of PDGFRβ in **(A)**. ***p < 0.001 (7, 14 dpi vs. pre and 3 dpi). **(E)** Western blot was used to detect the expression levels of PDGFB in M0, M1 or M2 macrophages (*n* = 5 per group). **(F)** Quantitative analysis of PDGFB in **(E)**, the blots were quantified as previously described, **p < 0.01 (M2 vs. M0), ^###^ *p* < 0.001 (M2 vs. M1). **(G)** ELISA was used to detect the concentration of PDGFB in CM0, CM1 or CM2 (*n* = 5 per group), The results were expressed as mean ± SD. **p < 0.01 (CM2 vs. other groups).

PDGFRβ is a specific marker for PDGFRβ^+^ pericytes while PDGFB can activate pericyte surface PDGFRβ and regulate pericyte migration in the retinal tissue. Therefore, we further investigated whether PDGFB/PDGFRβ pathway also plays an important role in the process of M2 macrophages promoting PDGFRβ^+^ pericyte migration. Our results showed that the expression level of PDGFB increased significantly at 3 and 7 dpi compared with Pre (*p* < 0.01 and *p* < 0.05, respectively), while decreased significantly at 14 dpi compared with 3 and 7 dpi (*p* < 0.001 and *p* < 0.01, respectively, Figures 6A,B). The expression level of PDGFD was significantly decreased at 3, 7, and 14 dpi compared with Pre (*p* < 0.0001, Figures 6A,C). In addition, the expression level of PDGFRβ was increased significantly at 7 and 14 dpi compared with Pre and 3 dpi (*p* < 0.001, Figures 6A,D), consistent with immunofluorescence staining that PDGFRβ^+^ pericytes aggregated to the injury core (Figure 1). These preliminarily suggested that PDGFB rather than PDGFD may be involved in PDGFRβ^+^ pericytes migration after SCI.

Secondly, the expression level of PDGFB was detected after macrophage polarization *in vitro*. The results of Western blot showed that the expression level of PDGFB in M2 macrophages was significantly higher than that in other groups (*p* < 0.01 and *p* < 0.001, respectively, Figures 6E,F). Besides, the results of ELISA showed that the concentration of PDGFB in M2 macrophages culture medium was significantly higher than that in other groups (*p* < 0.01, Figure 6G). These furtherly suggested that PDGFB may be involved in M2 macrophages promoting PDGFRβ^+^ pericytes migration after SCI.

### M2 Macrophages Secrete PDGFB Acting on PDGFRβ to Promote PDGFRβ^+^ Pericytes Migration *in Vitro*

**FIGURE 7:**
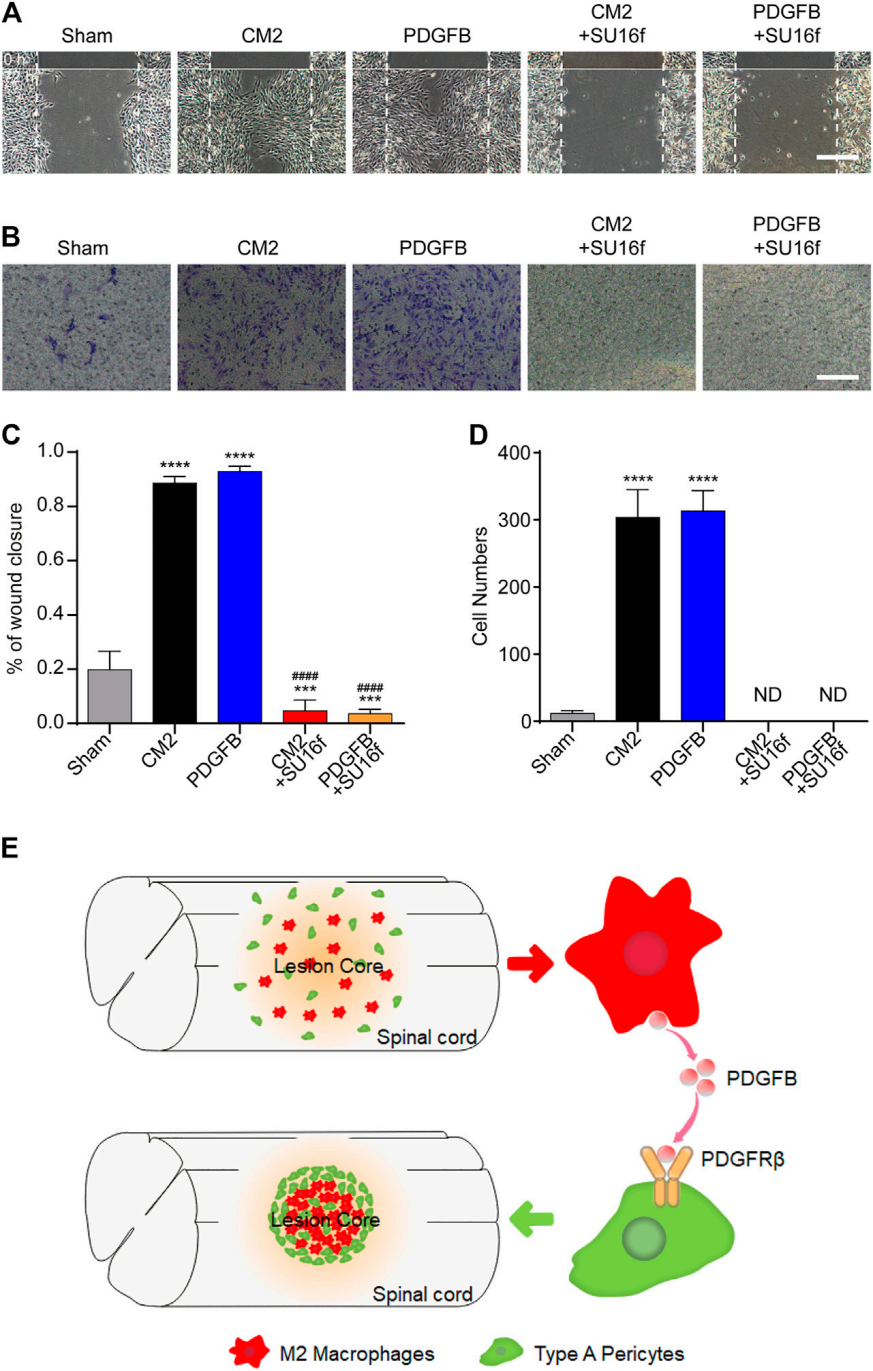
M2 macrophages secrete PDGFB acting on PDGFRβ to promote PDGFRβ^+^ pericytes migration *in vitro*. **(A)** Scratch test was used to detect the migration of PDGFRβ^+^ pericytes after being treated with DMEM (Sham), conditioned medium of M2 macrophages (CM2), 10 ng/ml recombinant mouse PDGFB protein (PDGFB), CM2 plus 10 μm PDGFRβ inhibitor (CM2+SU16f) or PDGFB plus 10 μM PDGFRβ inhibitor (PDGFB+SU16f) for 72 h (*n* = 3 per group). **(B)** Transwell test was used to further detect the migration of PDGFRβ^+^ pericytes after being treated in Sham, CM2, PDGFB, CM2 + SU16f or PDGFB+SU16f groups for 20 h (*n* = 3 per group). **(C)** Quantitative analysis of the wound closure rate in **(A)**, the results were quantified as previously described, ****p < 0.0001, ***p < 0.001 (groups vs. Sham), ^####^ *p* < 0.0001 (groups vs. CM2 and PDGFB). **(D)** Quantitative analysis of the number of transmembrane cells in **(B)**, the results were quantified as previously described, ****p < 0.0001 (CM2 and PDGFB vs. other groups). ND, not determined. Scale bars, 100 μm. **(E)** Schematic representation. M2 macrophages could secrete PDGFB, acting on PDGFRβ of PDGFRβ^+^ pericytes, which promote the formation of fibrotic scar, corral macrophages and limit inflammation after SCI.

To investigate the effect of M2 macrophages secretion of PDGFB on PDGFRβ^+^ pericytes migration and its possible mechanism, SU16f (Andersen et al., 2015; Jiang et al., 2017; Chatterjee et al., 2019) was used to specifically inhibit PDGFRβ and then the effect of exogenous PDGFB and M2 macrophages conditioned medium on PDGFRβ^+^ pericytes migration were observed *in vitro*. The scratch test showed that compared with Sham group, M2 conditioned medium and exogenous PDGFB significantly promoted the wound closure rate of pericytes (*p* < 0.0001) while the promotion effect was obviously eliminated by SU16f (*p* < 0.0001, Figures 7A,C). Besides, transwell test also showed that after adding M2 conditioned medium or exogenous PDGFB, the number of pericytes passing through membrane increased significantly compared with other groups while the promotion effect was significantly eliminated by SU16f (*p* < 0.0001, Figures 7B,D). These suggested that M2 macrophages promote PDGFRβ^+^ pericytes migration *via* PDGFB/PDGFRβ pathway *in vitro.*

## Discussion

At present, there is no effective treatment for SCI clinically, and investigating the pathological changes of SCI is expected to provide new ideas and therapeutic targets (Selvarajah et al., 2015; Bellver-Landete et al., 2019; Kobayakawa et al., 2019). After SCI, oxidative stress caused by ischemia-reperfusion injury and inflammatory cytokines released by macrophages lead to the cascade of inflammatory responses (Hausmann, 2003; David and Kroner, 2011; Muradov et al., 2013; Tran et al., 2018). Fibrotic scar surrounds macrophages to prevent inflammation from spreading while finally inhibits axonal regeneration (Klapka et al., 2005; Göritz et al., 2011; Dias et al., 2018). It has been reported that reducing of fibrotic scar formation could promote nerve regeneration and functional recovery (Klapka et al., 2005; Dias et al., 2018). However, there are no specific drugs to eliminate fibrotic scar after SCI, and the mechanism of fibrotic scar formation is still unclear. Therefore, the study uncovering the mechanism of fibrotic scar formation after SCI is expected to provide new ideas and therapeutic targets.

Using genetic lineage tracing, Göritz et al. (2011) first identified a subset of pericytes as the source of the fibrotic scar after SCI in 2011, which was further confirmed by Jae K. Lee et al. (Soderblom et al., 2013). This subset of pericytes forming fibrotic scar express PDGFRβ and CD13 as molecular markers while PDGFRβ was expressed in all this subset of pericytes after SCI and can be used as a specific marker (Göritz et al., 2011; Soderblom et al., 2013; Dias et al., 2018). PDGFRβ^+^ pericytes are mainly distributed around large diameter vessels in the normal spinal cord. After SCI, PDGFRβ^+^ pericytes and macrophages gradually migrate and aggregate to the injury core (Göritz et al., 2011; Soderblom et al., 2013). Finally, PDGFRβ^+^ pericytes and extracellular matrix including Collagen, Fibronectin and Laminin surround the macrophages to form fibrotic scar, which prevents nerve regeneration (Klapka et al., 2005; Göritz et al., 2011; Dias et al., 2018). The mechanism by which PDGFRβ^+^ pericytes migrate and aggregate to the injury core is unclear. The spatiotemporal distribution of macrophages and PDGFRβ^+^ pericytes is closely related after SCI, and the clearance of blood-derived macrophages resulted in the reduction of fibrotic scar in the injury core after SCI (Zhu et al., 2015a). These suggest that macrophages may be involved in the migration and aggregation of PDGFRβ^+^ pericytes after SCI, which needs to be confirmed by further experiments. Mac2 has been shown to be a specific marker for blood-derived macrophages after SCI (Wang et al., 2015). Therefore, we used anti-Mac2 antibody to specifically label macrophages rather than microglia and observed the spatiotemporal distribution of macrophages and PDGFRβ^+^ pericytes after SCI. The results showed that macrophages and PDGFRβ^+^ pericytes gradually migrated and aggregated to the injury core at 3–7 dpi while PDGFRβ^+^ pericytes surrounded macrophages and formed fibrotic scar at 14 dpi, consistent with the conclusions of Jae K. Lee et al. (Zhu et al., 2015a). These further suggested that macrophages may be involved in the migration and aggregation of PDGFRβ^+^ pericytes after SCI.

Macrophages play different roles due to M1 or M2 polarization in many diseases, which is important for oxidative stress, immune response and homeostasis repair (Kigerl et al., 2009; David and Kroner, 2011; Hu et al., 2015; Wang et al., 2015; Wang et al., 2019). M1 macrophages are pro-inflammatory phenotypes with high expression of iNOS, CD16/32 and TNF-α, and have the functions of phagocytosis and clearance of pathogens. M2 macrophages are anti-inflammatory phenotypes, with high expression of CD206, Arg-1 and PDGF, and have the functions of growth stimulation, tissue repair and Collagen formation (Kigerl et al., 2009; David and Kroner, 2011). However, the effect and mechanism of macrophages polarization on the migration of PDGFRβ^+^ pericytes to the injury core after SCI have not been reported. It has been reported that after SCI, most macrophages/microglial cells are M1 phenotype, with only a transient number showing M2 phenotype which occurs at 3–7 dpi (Kigerl et al., 2009). Blood-derived macrophages can be distinguished from microglia based on the expression of Mac2, which provides a more accessible tool of studying macrophage polarization in addition to transgenic mice after SCI (Wang et al., 2015). Therefore, in our study, Mac2^+^ iNOS^+^ and Mac2^+^ CD206^+^ were used to label M1 and M2 macrophages, respectively. Our results showed that at 3–7 dpi, PDGFRβ^+^ pericytes gradually migrated and aggregated to the injury core, while macrophages were mainly M2 phenotype at this time. At 14 dpi, PDGFRβ^+^ pericytes had surrounded macrophages to form fibrotic scar, while macrophages were mainly M1 phenotype at this time. Our results on the polarization of macrophages after SCI are consistent with the study from Kigerl et al. (2009). These suggested that M2 macrophages may be involved in the migration and aggregation of PDGFRβ^+^ pericytes after SCI.

To further investigate the effect and mechanism of M2 macrophages on PDGFRβ^+^ pericytes migration after SCI, RAW 264.7 cells and MBVP cells were used for *in vitro* studies. The scratch test and transwell test showed that after adding M2 conditioned medium, the migration ability of PDGFRβ^+^ pericyte was significantly enhanced. These suggested that M2 macrophages could promote PDGFRβ^+^ pericytes migration *in vitro*, and this effect of promoting migration may be due to exocrine of certain cytokines by M2 macrophages.

PDGFRβ, as a specific marker of PDGFRβ^+^ pericytes, is a receptor subtype of the PDGF family (Klinkhammer et al., 2018; Sil et al., 2018). It has been reported that only PDGFB and PDGFD can activate PDGFRβ and play important roles in cell migration and differentiation (Evrova and Buschmann, 2017). Moreover, Dubrac et al. (2018) showed that PDGFB can activate pericyte surface PDGFRβ to regulate pericyte migration in the retinal tissue. However, the role of PDGFB/PDGFRβ pathway in PDGFRβ^+^ pericytes migration after SCI has not been reported. Our results showed that the expression of PDGFB increased significantly at 3 dpi, reached its peak at 7 dpi, while decreased significantly at 14 dpi. The expression of PDGFD was significantly decreased at 3, 7, and 14 dpi. These suggested that PDGFB may be involved in the migration of PDGFRβ^+^ pericytes after SCI. Besides, the expression of PDGFB was significantly increased after M2 polarization of macrophages *in vitro*, which led us to speculate that M2 macrophages may activate PDGFRβ by secreting PDGFB, promoting PDGFRβ^+^ pericytes migration after SCI. To test this hypothesis, we used SU16f to specifically block pericytes surface PDGFRβ (Andersen et al., 2015; Jiang et al., 2017; Chatterjee et al., 2019) and observed the effect of M2 macrophage conditioned medium or exogenous PDGFB on PDGFRβ^+^ pericyte migration *in vitro*. The scratch test and transwell test showed that after adding M2 conditioned medium, PDGFRβ^+^ pericyte migration ability was significantly enhanced, but this promotion effect could be blocked by SU16f. These suggested that M2 macrophages secrete PDGFB acting on PDGFRβ to promote PDGFRβ^+^ pericytes migration after SCI (Figure 7E).

Our study also showed that M2 macrophages could promote PDGFRβ^+^ pericytes secretion of extracellular matrix such as Laminin, but the molecular mechanism needs to be further studied. Although PDGFRβ was expressed in all the subset of pericytes forming fibrotic scar after SCI, a small number of PDGFRβ^+^ pericytes were distributed around large diameter vessels. Therefore, studies of SU16f as a PDGFRβ specific inhibitor should be evaluated for its side effects on angiogenesis and vascular function. In addition, systematic *in vivo* experiments are needed to further confirm our conclusions.

In conclusion, the present study demonstrated that macrophages are closely related to the spatiotemporal distribution of PDGFRβ^+^ pericytes in the injury core at 3, 7, and 14 dpi. Macrophages appear M2 polarization at 3 and 7 dpi while M1 polarization at 14 dpi. M2 macrophages can promote PDGFRβ^+^ pericytes migration *via* PDGFB/PDGFRβ pathway, which can be blocked by a PDGFRβ specific inhibitor SU16f. Besides, M2 macrophages can promote PDGFRβ^+^ pericytes to secrete extracellular matrix. These suggested that PDGFB/PDGFRβ pathway is a promising new target for the treatment of SCI.

## Data Availability

The original contributions presented in the study are included in the article/Supplementary Material, further inquiries can be directed to the corresponding authors.
